# Role of PD-L1 in licensing immunoregulatory function of dental pulp mesenchymal stem cells

**DOI:** 10.1186/s13287-021-02664-4

**Published:** 2021-12-04

**Authors:** Rosanna Di Tinco, Giulia Bertani, Alessandra Pisciotta, Laura Bertoni, Elisa Pignatti, Monia Maccaferri, Jessika Bertacchini, Paola Sena, Antonio Vallarola, Rossella Tupler, Stefania Croci, Martina Bonacini, Carlo Salvarani, Gianluca Carnevale

**Affiliations:** 1 Department of Surgery, Medicine Dentistry and Morphological Sciences with Interest in Transplant, University of Modena and Reggio Emilia, Modena, Italy; 2 Department of Biomedical, Metabolic and Neural Sciences, Center for Neuroscience and Neurotechnology, University of Modena and Reggio Emilia, Modena, Italy; 3 Clinical Immunology, Allergy and Advanced Biotechnologies Unit, Azienda Unità Sanitaria Locale-IRCCS di Reggio Emilia, Reggio Emilia, Italy; 4 Rheumatology Unit, Azienda Unità Sanitaria Locale-IRCCS di Reggio Emilia, Reggio Emilia, Italy

**Keywords:** DPSCs, Immunomodulatory functions, PD1/PD-L1 pathway, Neural crest stem cells

## Abstract

**Background:**

Dental pulp stem cells (DPSCs) are low immunogenic and hold immunomodulatory properties that, along with their well-established multi-potency, might enhance their potential application in autoimmune and inflammatory diseases. The present study focused on the ability of DPSCs to modulate the inflammatory microenvironment through PD1/PD-L1 pathway.

**Methods:**

Inflammatory microenvironment was created in vitro by the activation of T cells isolated from healthy donors and rheumatoid arthritis (RA) patients with anti-CD3 and anti-CD28 antibodies. Direct and indirect co-cultures between DPSCs and PBMCs were carried out to evaluate the activation of immunomodulatory checkpoints in DPSCs and the inflammatory pattern in PBMCs.

**Results:**

Our data suggest that the inflammatory stimuli trigger DPSCs immunoregulatory functions that can be exerted by both direct and indirect contact. As demonstrated by using a selective PD-L1 inhibitor, DPSCs were able to activate compensatory pathways targeting to orchestrate the inflammatory process by modulating pro-inflammatory cytokines in pre-activated T lymphocytes. The involvement of PD-L1 mechanism was also observed in autologous inflammatory status (pulpitis) and after direct exposure to pre-activated T cells from RA patients suggesting that immunomodulatory/anti-inflammatory properties are strictly related to their stemness status.

**Conclusions:**

Our findings point out that the communication with the inflammatory microenvironment is essential in licensing their immunomodulatory properties.

**Supplementary Information:**

The online version contains supplementary material available at 10.1186/s13287-021-02664-4.

## Background

Human dental pulp is considered an interesting source of adult stem cells, due to the low-invasive isolation procedures, high content of stem cells and its peculiar embryological origin from neural crest. During embryogenesis, neural crest stem cells migrate into different developing structures and give rise to a plethora of cells and tissues and thus are considered the prime contributors in the formation of neural, skeletal and mesenchymal structures of the craniofacial complex. Human dental pulp stem cells (DPSCs) are localized in the perivascular area, express mesenchymal stem cells (MSCs) phenotypic markers, exert multipotency properties and are characterized by low immunogenicity and immunomodulatory properties [1]. Despite the paucity of clinical trials, DPSCs can differentiate in vitro and in vivo into Schwann cells and participate in peripheral nerve regeneration [2], contribute to restore urethral sphincter contractile function [3], reduce fibrosis and ameliorate muscle trophism in Duchenne Muscular Dystrophy in SCID/mdx mouse model [4]. As previously suggested, MSCs should be instead referred to as “Medicinal Signaling Cells” because they serve as reservoir/carrier of bioactive molecules with immunomodulatory and trophic activities [5]. It is well established that MSCs not only represent a source of stem cells with regenerative potential but also modulate the inflammatory response and empower other cells to participate in tissue homeostasis and regeneration [6–8]. Their immunomodulatory/anti-inflammatory potential has generated an emerging multidisciplinary research field that translates MSC-based therapies to the clinic for the treatment of inflammatory and autoimmune diseases [9–11]. As previously demonstrated, DPSCs are able to trigger lymphocytes apoptosis by activating the Fas/FasL pathway [12–14]. Moreover, DPSCs exert immunomodulatory properties even through the modulation of pro-inflammatory cytokines release when exposed to activated peripheral blood mononuclear cells (PBMCs) obtained from COVID-19 patients [15, 16]. To this regard, the immunomodulatory/anti-inflammatory ability of DPSCs can be induced by the inflammatory microenvironment itself shaping a plastic immunosuppressive potential [17, 18]. As a matter of fact, DPSCs might take advantage of different immunomodulatory mechanisms through different surface proteins when exposed to an inflammatory microenvironment. Among these surface proteins, Programmed cell-death ligand 1 (PD-L1) seems to be an important effector in the suppression of immune system activation and its role is not fully understood in DPSCs [19–21]. PD1/PD-L1 pathway is physiologically involved in the establishment and maintenance of immunological tolerance, moreover Programmed cell death 1 (PD1) is required to maintain stem cells properties in DPSCs [22]. PD1 and PD-L1 can be expressed on activated CD4^+^ and CD8^+^ T cells, B cells, monocytes, NK cells and dendritic cells [23]. The checkpoint blockade and the alteration of PD1/PD-L1 pathway might be the basis for the development of inflammatory autoimmune diseases [24–27]. Based on the important role of PD1/PD-L1 signaling in autoimmunity, this pathway has become a new therapeutic target for ameliorating autoimmune diseases by increasing the expression of PD-L1 or triggering PD1. In light of these observations, the aim of our study is to investigate the ability of DPSCs to activate PD1/PD-L1 pathway and how their immunomodulatory abilities can be affected by simulating an inflammatory microenvironment in vitro and under physiological inflammatory conditions.

## Methods

### Dental pulp stem cells isolation and characterization

Human DPSCs were isolated from third molars of adult subjects (*n* = 3; 18–25 years) undergoing routine dental extraction. All subjects gave written informed consent in accordance with the Declaration of Helsinki. The study was performed according to the recommendations of Comitato Etico Provinciale-Azienda Ospedaliero-Universitaria di Modena (Modena, Italy), which approved the experimental protocol (Ref. Number 3299/CE; 5 September 2017). Briefly, dental pulp was harvested from the teeth and enzymatically digested by immersion in a digestive solution containing 3 mg/ml type I collagenase plus 4 mg/ml dispase. In order to obtain a cell suspension pulp was filtered onto 100 µm Falcon Cell Strainers. Following cell expansion in culture medium (a-MEM supplemented with 10% heat inactivated fetal bovine serum (FBS), 2 mM L-glutamine, 100 U/ml penicillin, 100 mg/ml streptomycin; all from Sigma Aldrich, St. Louis, MO, United States) at 37° C and 5% CO_2_, immune-selection was performed on DPSCs by using MACS separation kit (Miltenyi Biotec, Bergisch Gladbach, Germany), according to manufacturer’ instructions with the following primary antibodies: mouse IgM anti-STRO-1 and rabbit IgG anti-c-Kit (Santa Cruz Biotechnology, Dallas, TX, United States). The following magnetically labeled secondary antibodies were used: anti-mouse IgM and anti-rabbit IgG (Miltenyi Biotec). The selection and immunophenotypical characterization of STRO-1^+^/c-Kit^+^ DPSCs at passage 1 was then evaluated by immunofluorescence and fluorescence activated cell sorting (FACS) analyses.

### Osteogenic differentiation of DPSCs and BM-MSCs

Cells were seeded at 3 × 10^3^ cells/cm^2^ in osteogenic medium consisting in culture medium supplemented with 5% FBS, 100 μM 2P-ascorbic acid, 100 nM dexamethasone, 10 mM β-glycerophosphate for 3 weeks. Osteogenic commitment of DPSCs was evaluated through Alizarin Red stain to assess mineralized extracellular matrix deposition. Differentiated DPSCs were fixed in 4% paraformaldehyde (PFA) and incubated for 5 min at room temperature in a solution containing 0.1% alizarin red and 1% ammonium hydroxide. In parallel, osteogenic differentiation of BM-MSCs was also performed.

### Isolation of human amniotic fluid stem cells and bone-marrow derived mesenchymal stem cells

Supernumerary amniocentesis samples (*n* = 3) were provided by Genetics laboratory of IRCCS Arcispedale Santa Maria Nuova, Reggio Emilia, Italy. All the samples were collected with the written informed consent of the patients, in accordance with Italian law and ethical committee guidelines. Human amniotic fluid stem cells (AFSCs) were isolated as previously described [4]. Briefly, human amniocentesis cultures were harvested by trypsinization and immunoselected by MACS with a rabbit IgG anti-c-Kit Ab (Santa Cruz Biotechnology). Human bone marrow derived mesenchymal stem cells (BM-MSCs) were isolated from bone specimens obtained from the femoral heads of male patients (*n* = 3, 18–21 years) undergoing total orthopedic surgical procedures. Informed consent was obtained according to Italian law and the guidelines of the ethical committee of IRCCS Arcispedale Santa Maria Nuova, Reggio Emilia, Italy. In particular, bone tissue consisting mainly of trabecular elements dissociated from the cortical bone was mechanically reduced to small fragments and incubated in standard culture conditions. Adherent cells outgrown from bone fragments were expanded and immunophenotypically characterized for the typical MSC markers [28]. AFSCs and BM-MSCs were expanded in standard culture medium and used for subsequent evaluations at passage 1.

### PBMCs isolation and co-culture with DPSCs

Human peripheral blood was collected from healthy donors (*n* = 5) and rheumatoid arthritis (RA) patients (*n* = 3) who gave written informed consent, according to the guidelines of the ethics committee. PBMCs were isolated by using Histopaque (Sigma Aldrich), according to the manufacturer’ instructions, and pre-activated as previously described [12]. In particular, direct co-culture systems were established by seeding DPSCs, AFSCs and BM-MSCs at a cell density of 5000 cells/cm^2^ and cultured in RPMI 1640 medium supplemented with 10% FBS, 2 mM glutamine, 100 units/ml penicillin and 100 mg/ml streptomycin. Upon stem cells adhesion, after 24 h, PBMCs (resting or pre-activated according to each experimental evaluation; rPBMCs, aPBMCs) were seeded on stem cells at a 10:1 ratio and kept in culture for 72 h in presence or absence of a specific PD-L1 inhibitor (10 μg/ml; Thermo Fisher, Waltham, MA, USA) [29]. Likewise, transwell culture system was set up by using 0.4 µm transwell inserts (Corning, New York, NY, USA) under the same seeding conditions. At the end of the co-culture, the floating PBMCs together with supernatant and the adherent DPSCs, AFSCs and BM-MSCs were removed and collected separately, for subsequent experimental procedures. DPSCs, AFSCs, BM-MSCs and PBMCs cultured alone were used as controls.

### Western blot and immunoprecipitation analyses

Western blot analyses were performed on whole cell lysates as formerly described [12]. Briefly, 30 µg of protein extract per sample were quantified by a Bradford Protein Assay (Bio-Rad, Hercules, CA, USA), then SDS–polyacrylamide gel electrophoresis and subsequent protein transfer to nitrocellulose membranes were carried out. The following primary antibodies were used: rabbit anti-PD-L1 (Novus Biologicals, Centennial, CO, USA), mouse anti-PD1 (Invitrogen, Waltham, MA, Stati Uniti), rabbit anti-FasL (Santa Cruz Biotechnology), mouse anti-PCNA (Merck Millipore, Burlington, MA, USA), rabbit anti-caspase 3 (Santa Cruz Biotechnology), mouse anti-osteopontin (OPN; Abcam, Cambridge, UK) diluted 1:1000 in Tris buffered saline Tween 20 with 2% bovine serum albumin (BSA) and 3% non-fat powder milk. Membranes were then incubated with HRP-conjugated anti-mouse and anti-rabbit secondary antibodies (Thermo Fisher, Waltham, MA, USA) diluted 1:2000, for 1 h at room temperature. The immunoblotting was revealed using Clarity™ Western ECL Substrate (Bio-Rad Laboratories). Mouse anti-actin antibody (Santa Cruz Biotechnology) was used as a control of protein loading. Standard control (SC) for cleaved caspase-3 was purchased from Cell Signalling Technology. Fiji ImageJ software was used to perform densitometry analysis. An equal area was selected inside each band and the mean of gray levels (in a 0–256 scale) was calculated. Data were then normalized to values of background and of control actin band. Immunoprecipitation assays for PD1 and PD-L1 proteins were performed by Immunoprecipitation Starter Pack according to the manufacturers’ instructions (GE Healthcare, Little Chalfont, Buckinghamshire, UK). Three hundred µg of extracts were pre-cleared with 20 µl of protein A/G, for 1 h at 4 °C and incubated with 2 µg of mouse monoclonal anti-human PD1 antibody (Abcam) or 2 µg of rabbit polyclonal anti-human PD-L1 antibody (Novus Biologicals) respectively, for 1 h at 4 °C. Then, 50 µl of protein A/G, previously equilibrated in the same extraction buffer, were added, and incubated overnight at 4 °C. After three washing steps, the bound protein was detached from the resin with SDS loading buffer, recovered by centrifugation and revealed by Western blot, as described above. Extracts immunoprecipitated by using a mouse and a rabbit anti-human CD95 antibody (Cell Signaling Technology) were used as negative control of PD1 and PD-L1 proteins.

### Confocal immunofluorescence analyses

Cells were fixed with 4% PFA in pH 7.4 phosphate buffer saline (PBS) for 20 min and washed in PBS. If necessary, an incubation for 5 min with Triton-X 100 0.1% in PBS was performed in order to obtain the membrane permeabilization. After rinsing with PBS, samples were blocked with 3% BSA in PBS for 30 min at room temperature and then incubated with the following primary antibodies: mouse IgM anti-STRO-1, rabbit anti-c-Kit (Santa Cruz Biotechnology), mouse anti-CD3 (Miltenyi Biotec), rabbit anti-PD-L1 (Novus Biologicals), mouse anti-osteocalcin (OCN; Abcam), rabbit anti-RUNX2 (Abcam), rabbit anti-FasL (Santa Cruz Biotechnology), mouse anti-nestin (Merck Millipore), rabbit anti-Ki-67 (Cell Signalling Technology) all diluted 1:100 in PBS containing 1% BSA, for 1 h at room temperature. After washing in PBS containing 1% BSA, the samples were incubated for 1 h at room temperature with the secondary antibodies diluted 1:200 in PBS containing 1% BSA (goat anti-mouse Alexa488, goat anti-rabbit Alexa546, goat anti-mouse Alexa546, goat anti-rabbit Alexa488; Invitrogen). After washing with PBS, cells nuclei were stained with 1 μg/ml 4',6-Diamidino-2-Phenylindole Dihydrochloride (DAPI) in PBS for 10 min; then, samples were mounted with anti-fading medium (FluoroMount, Sigma-Aldrich). As formerly described, the multi-labelling immunofluorescence experiments were carried out avoiding cross-reactions between primary and secondary antibodies. Samples were observed by a Nikon A1 confocal laser scanning microscope. The confocal serial sections were processed with ImageJ software to obtain three-dimensional projections, and image rendering was performed using Adobe Photoshop Software. Cell proliferation was measured by counting the Ki-67^+^ and DAPI^+^ nuclei on 5 randomly selected fields measuring 1.0 × 10^5^ μm^2^ on 3 slides for each experimental group by a blind operator. Data were expressed as mean percentage ± SD of Ki-67^+^ DPSCs on DAPI labeled cells.

### Real time PCR analyses

Human PBMCs cells were homogenized, and total RNA was extracted and purified using the PureLink RNA columns (Thermo Fisher Scientific). cDNA synthesis was performed by using Maxima First Strand cDNA Synthesis Kit with DNase I treatment (Thermo Fisher Scientific). Quantitative real-time PCRs were performed using SYBR Green Master mix (Bio-Rad Laboratories) on CFX Connect Real-time PCR instrument (Bio-Rad Laboratories), with the following oligonucleotides: hRPLP0 (F: TACACCTTCCCACTTGCTGA, R: CCATATCCTCGTCCGACTCC); hIL-2 (F: AAAGAAAACACAGCTACAACTGG, R: GAAGATGTTTCAGTTCTGTGGC); hIFNγ (F: GCATCGTTTTGGGTTCTCTTG R: AGTTCCATTATCCGCTACATCTG), hTNFα (F: ACTTTGGAGTGATCGGCC, R: GCTTGAGGGTTTGCTACAAC), hIL-6 (F: CCACTCACCTCTTCAGAACG, R: CATCTTTGGAAGGTTCAGGTTG), hFasL (F: AAAGGAGCTGAGGAAAGTGG; R: CATAGGTGTCTTCCCATTCCAG); hCCL5 (F: TGCCCACATCAAGGAGTATTTC, R: CCATCCTAGCTCATCTCCAAAG); hCXCL10 (F: CCTTATCTTTCTGACTCTAAGTGGC, R: ACGTGGACAAAATTGGCTTG); hIL-10 (F: CAGAGTGAAGACTTTCTTTCAAATG, R: CCTTTAACAACAAGTTGTCCAGC).

Relative quantification was calculated from the ratio between the cycle number (Ct) at which the signal crossed a threshold set within the logarithmic phase of the given gene and that of the reference hRPLP0. Mean values of the duplicate results of three independent experiments for each sample were used as individual data for 2^−ΔΔCt^ statistical analysis.

### Flow cytometry analyses

Immune-phenotypical characterization of DPSCs was performed by FACS analysis on immune-selected DPSCs at passage 1. The expression of the typical mesenchymal stem cells (MSCs) markers, i.e., CD73, CD90, CD105, CD34, CD45, HLA-DR, was evaluated. Cells were stained with the following fluorochrome-conjugated antibodies (Abs): anti-human-CD73-PE-CY7, -CD90-FITC, -CD105-APC, -CD45-PE and -HLADR-PE-CY7 (all from BD Biosciences, Franklin Lakes, NJ, USA), and -CD34-ECD (Beckman Coulter, Fullerton, CA, USA). A minimum of 10,000 cells per sample was acquired and analyzed by using the Attune Acoustic Focusing Flow Cytometer (Attune NxT, Thermo Fisher Scientific) Data was analyzed by FlowJo 9.5.7 (Treestar, Inc., Ashland, OR, USA). After 72 h of culture, supernatants containing PBMCs were collected and DPSCs were detached by the use of trypsin–EDTA. Cells were pelleted by centrifugation at 400×*g* for 5 min then washed once in PBS. Cells were suspended in 100 ul fixable near-IR dead cell stain (Invitrogen) at 0.1% in PBS and incubated at room temperature for 30 min. Cells were washed with PBS+ 1% FBS then suspended in 100 μL PBS+ 1% FBS containing the following antibodies: CD3-PerCP (clone BW264/56), CD56-PE (clone REA196), CD4-APC (clone REA623), PD1-FITC (clone EH12.2H7), PD-L1-PECy7 (clone 29E2A3) purchased from BioLegend (BioLegend, San Diego, CA, USA). Antibodies were used as suggested by the manufacturer. Cells were stained for 30 min at 4 °C. After washing, cells were resuspended in PBS+ 1% FBS and acquired with the FACSCanto II flow cytometer (BD Biosciences), equipped with two lasers for excitation at 488 and 633 nm. FBS used in flow cytometry assays was heat inactivated. Data were analyzed with FACSDiva 8.0.1 software. To determine if PD1 and PD-L1 were expressed on the cell surface, fluorescence minus one controls were used. Gating strategies to evaluate PD1 and PD-L1 expression by CD4^+^ T lymphocytes, CD4^−^ T lymphocytes and DPSCs are shown in Additional file 1.

### Immunohistochemical analyses

Immunohistochemistry was performed on 5 µm thick paraffin histological sections of healthy and pulpitis affected dental pulps (6 sections per sample), respectively. Immunolabeling was carried out by incubating with rabbit anti-PD-L1 (1:50; Novus Biologicals) primary antibody and subsequently with an anti-rabbit HRP-labelled secondary antibody (1:100; Thermo Fisher Scientific), both diluted in PBS containing 1% BSA. HRP was revealed by a 3,3’- Diaminobenzidine (DAB) based kit (Sigma Aldrich). Counterstaining was carried out through hematoxylin stain, then slides were mounted with Eukitt (Carlo Erba Reagents, Cornaredo, Italy).

### Statistical analysis

All the experiments were performed in triplicate. Data were expressed as Mean ± Standard Deviation (SD). Student t test was used to analyze differences among two experimental groups. One way ANOVA followed by Newman-Keuls or Tukey post-hoc tests was performed to analyze differences among three or more experimental groups (GraphPad Prism Software version 5 Inc., San Diego, CA, USA). In any case, *P* values lower than 0.05 were considered statistically significant.

## Results

### Dental pulp stem cells isolation and immune phenotype characterization

**Fig. 1:**
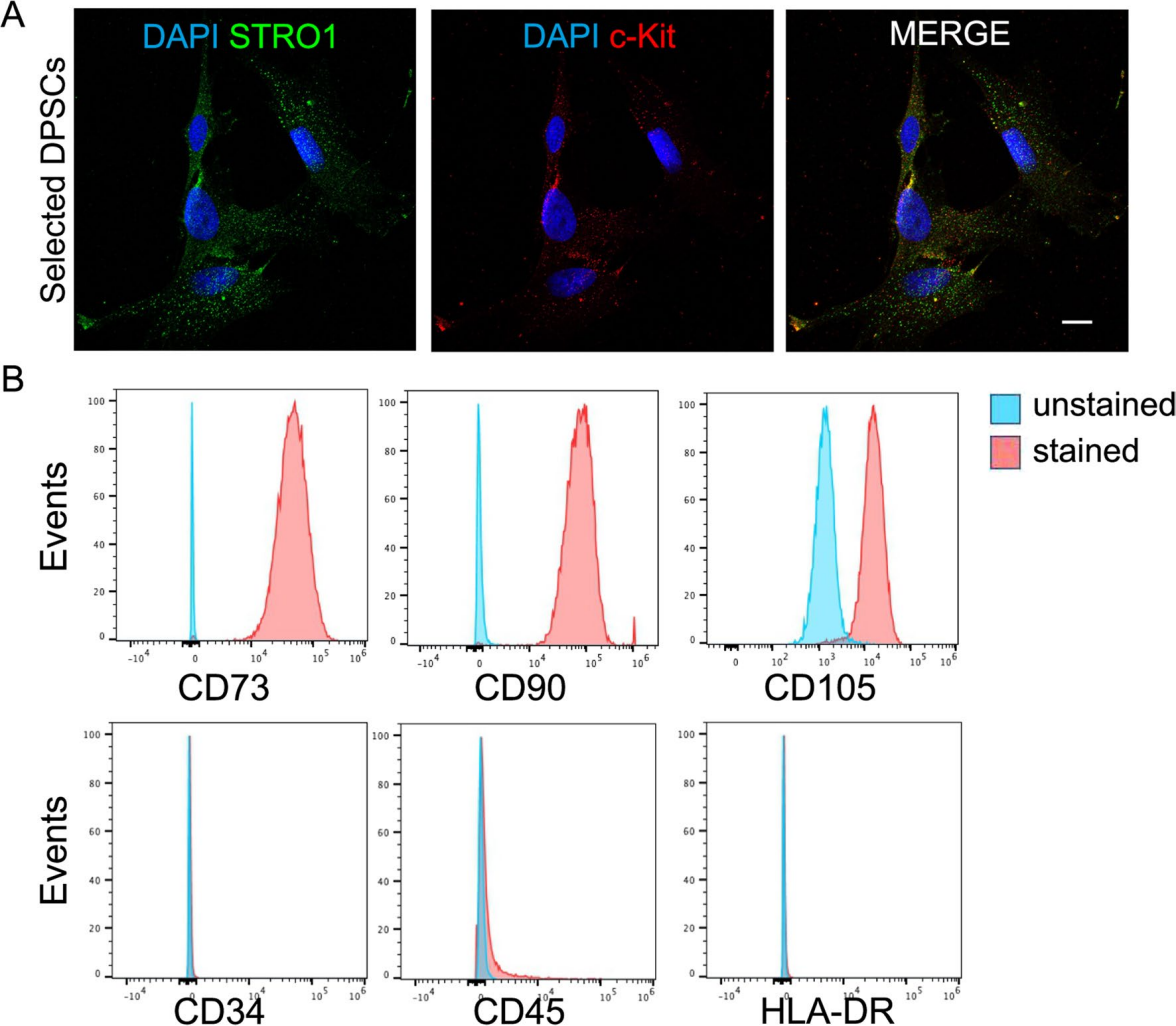
Dental pulp stem cells (DPSCs) characterization. **A** Immunofluorescence analysis on immune-selected DPSCs for stem cells markers STRO-1 (green) and c-Kit (red). Almost all selected cells are positively labeled against both stemness markers. Nuclei were counterstained with DAPI. Scale bar: 10 µm. **B** Immunophenotypical characterization and MSC markers expression in immune-selected DPSCs. DPSCs are 99% positive for CD73, CD90 and CD105 while being negative for CD34, CD45 and HLA-DR

After cell isolation and immune-selection, DPSCs positive stained for the expression of STRO-1 and c-Kit surface markers, as shown in Fig. 1A. Moreover, almost all immune-selected DPSCs (~ 99%) were positive stained for the expression of the typical MSC markers CD73, CD90 and CD105 whereas DPSCs were negative for the expression of CD34, CD45 and HLA-DR (Fig. 1B).

### Up-regulation of PD-L1 expression in DPSCs induced by activated PBMCs after direct co-culture

**Fig. 2:**
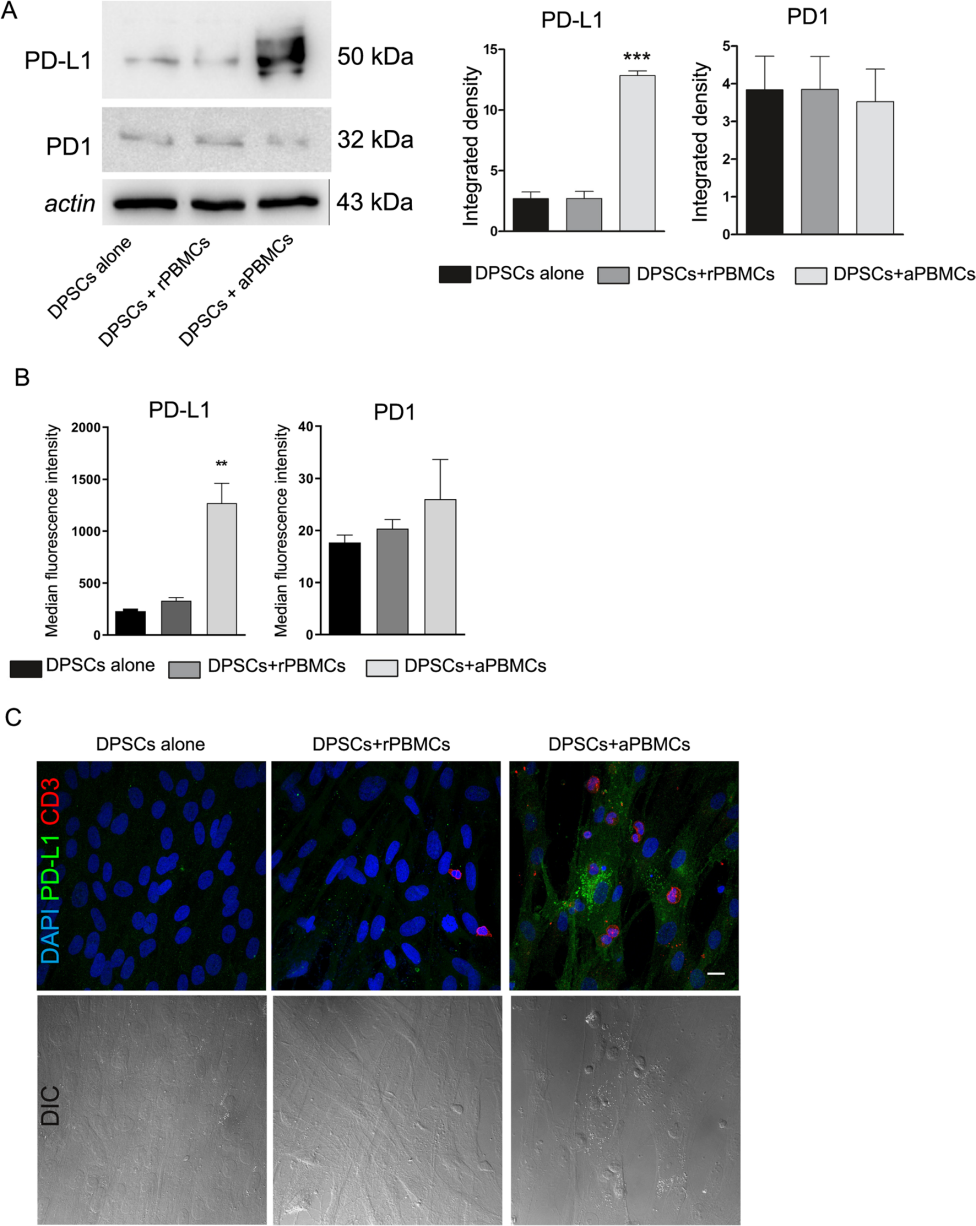
Immunomodulatory properties of DPSCs. **A** Western blot analyses performed on DPSCs alone and after direct co-culture with rPBMCs and aPBMCs for PD-L1 and PD1. Histograms show that DPSCs after co-culture with aPBMCs are able to express statistically significant higher levels of PD-L1. ***P < 0.001 versus DPSCs alone and versus DPSCs + rPBMCs. Data are expressed as mean ± SD and analyzed by one-way ANOVA followed by Newman-Keuls post-hoc test. **B** Expression of PD1 and PD-L1 was evaluated by flow cytometry on DPSCs cultured alone, co-cultured with rPBMCs and with aPBMCs. Median fluorescence intensity obtained by PD1 and PD-L1 staining minus median fluorescence intensity in FITC and PECy7 channels of FMO controls is shown. Data are represented as mean ± SD and analyzed by one-way ANOVA with Tukey post-hoc test. **C** Immunofluorescence analysis performed on DPSCs alone and in direct co-culture with rPBMCs and aPBMCs. DPSCs in co-culture with aPBMCs (red CD3+) are able to express PD-L1. DIC images on the bottom highlight the cell morphology and the co-culture system setup. Nuclei were counterstained with DAPI. Scale bar: 10 µm

PD1 and PD-L1 expression were evaluated in DPSCs after co-culture with rPBMCs and PBMCs pre-activated with anti-CD3 and anti-CD28, respectively. Western blot analyses revealed that PD-L1 expression was significantly up-regulated in DPSCs after direct co-culture with aPBMCs when compared to DPSCs cultured alone and after co-culture with rPBMCs (***P < 0.001 vs. DPSCs alone and DPSCs + rPBMCs; Fig. 2A). On the other hand, Western blot analyses showed that DPSCs expressed low levels of PD1 which were maintained even after co-culture with rPBMCs and aPBMCs (Fig. 2A). These data were confirmed by flow cytometry analysis, revealing that DPSCs showed low expression of PD1 and the co-culture with resting and pre-activated PBMCs did not induce any significant change of the cell surface marker (Gating strategy to determine PD1 and PD-L1 expression in DPSCs was reported in Additional file 1: Fig. S1). Up-regulation of PD-L1 in DPSCs after co-culture with aPBMCs was observed (**P < 0.01, DPSCs after co-culture with aPBMCs vs. DPSCs alone and vs. DPSCs after co-culture with rPBMCs; Fig. 2B).

These findings were supported by immunofluorescence analyses on DPSCs alone and directly co-cultured with resting and pre-activated PBMCs. In particular, as shown in Fig. 2C, only direct contact with aPBMCs (CD3^+^ lymphocytes stained in red) induced the expression of PD-L1 in DPSCs. DIC images reveal that co-culture system did not alter DPSCs morphology. Taken together these data indicated that only aPBMCs promote the activation of PD-L1 immunomodulatory checkpoint in DPSCs.

### Activation of PD1/PD-L1 pathway in stem cell niches and CD4^+^/CD4^−^ T lymphocytes

**Fig. 3:**
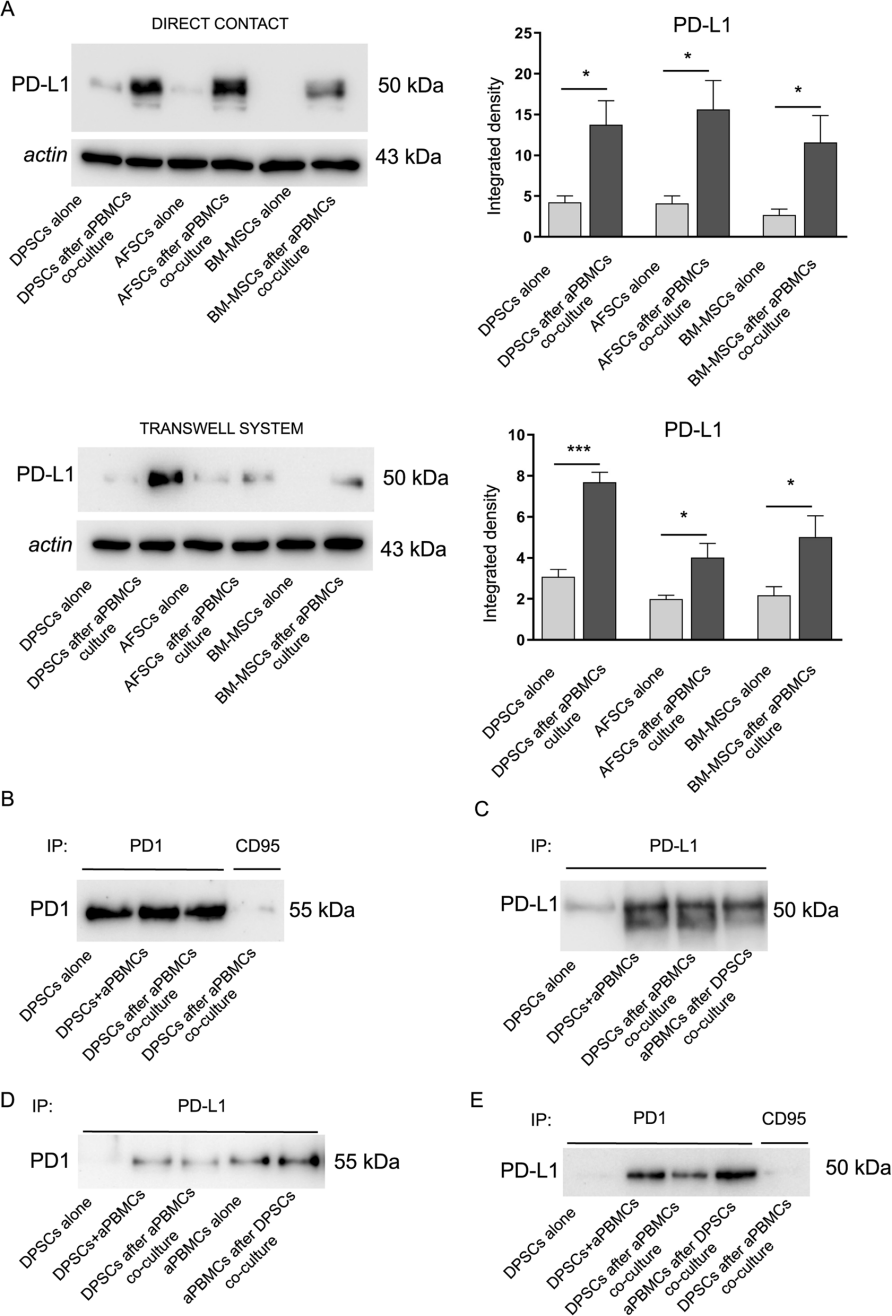
Activation of PD1/PD-L1 pathway in MSCs. **A** Western blot analyses of PD-L1 expression in DPSCs, AFSCs and BM-MSCs cultured alone and after co-culture with aPBMCs, either in direct co-culture conditions and transwell culture system. Histograms show that PD-L1 expression was statistically significant higher in MSCs after both direct co-culture and transwell culture conditions with aPBMCs. ***P < 0.001, *P < 0.05 versus MSCs cultured alone. Data are represented as mean ± SD and statistical analysis of differences between MSCs cultured alone and MSCs after co-culture was carried out by unpaired Student t test. **B–E** Human PD1 and PD-L1 proteins immunoprecipitation. **B** PD1 protein was immunoprecipitated and revealed with anti-PD1 antibody. **C** PD-L1 protein was immunoprecipitated and revealed with anti-PD-L1 antibody. **D** PD-L1 protein was immunoprecipitated with anti-PD-L1 antibody and revealed with anti-PD1 antibody. **E** PD1 protein was immunoprecipitated with anti-PD1 antibody and revealed with anti-PD-L1 antibody. CD95 antibody was used as negative control

The immunomodulatory promoting effect of aPBMCs was evaluated in different stem cell populations, including DPSCs, amniotic fluid stem cells (AFSCs) and bone marrow derived mesenchymal stem cells (BM-MSCs). PD-L1 expression was evaluated in stem cells after direct co-culture and indirect (i.e. transwell) culture conditions. Western blot analyses revealed that DPSCs, AFSCs and BM-MSCs expressed statistically significant higher levels of PD-L1 after 72 h of either direct co-culture and transwell culture conditions with aPBMCs, when compared to the same stem cell populations cultured alone (***P < 0.001, *P < 0.05 vs. stem cell populations cultured alone; Fig. 3A). These data indicate that PD-L1 is an immunomodulatory effector, whose expression is shared by different stem cell populations following both direct co-culture with aPBMCs and transwell culture conditions through the release of soluble factors.

The interaction between PD-L1 and PD1 in DPSCs and aPBMCs was evaluated by immunoprecipitation analysis (Fig. 3B–E). The technical control of successful immunoprecipitation procedure was reported in Fig. 3B and C. Immunoprecipitating samples for PD1, PD-L1 and revealing for the same markers respectively, we confirmed that aPBMCs and DPSCs express both PD1 and PD-L1 (Fig. 3B, C).

**Fig. 4:**
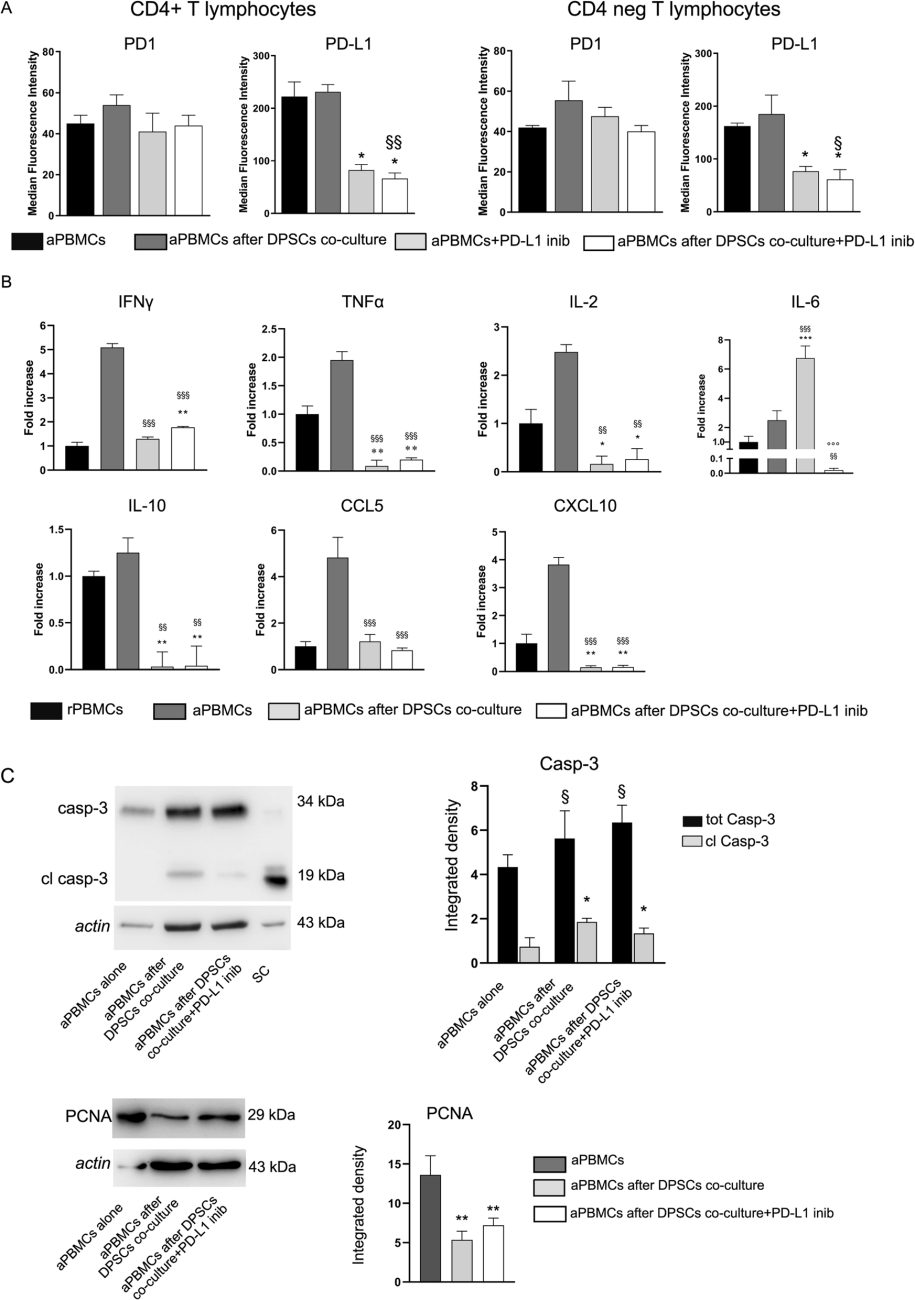
Evaluation of PD1/PD-L1 pathway and inflammatory cytokines expression in aPBMCs after DPSCs co-culture. **A** Expression of PD1 and PD-L1 was evaluated by flow cytometry on aPBMCs cultured alone and co-cultured with DPSCs with or without anti-PD-L1 blocking antibody. Median fluorescence intensity obtained by PD1 and PD-L1 staining minus median fluorescence intensity in FITC and PECy7 channels of FMO controls is shown in CD4^+^ and CD4^−^ gated T lymphocytes. Data are expressed as mean ± SD and analyzed by one-way ANOVA followed by Tukey post-hoc test. *P < 0.05 aPBMCs + PD-L1 inib, aPBMCs after DPSCs co-culture + PD-L1 inib vs. aPBMCs; ^§^*P* < 0.05, ^§§^*P* < 0.01 aPBMCs after DPSCs co-culture + PD-L1 inib vs. aPBMCs after DPSCs co-culture. **B** The expression of IFNγ, TNFα, IL-2, IL-6, IL-10, CCL5 and CXCL10 was evaluated through Real Time PCR analyses on rPBMCs and aPBMCs alone, aPBMCs after DPSCs co-culture with and without PD-L1 selective inhibitor. Data are expressed as mean ± SD and analyzed by one-way ANOVA followed by Newman-Keuls post-hoc test. *P < 0.05, **P < 0.01, ***P < 0.001 vs. rPBMCs; ^§§^*P* < 0.01, ^§§§^*P* < 0.001 vs. aPBMCs; °°°*P* < 0.001 vs. aPBMCs after DPSCs co-culture. **C** The expression of caspase 3 and PCNA on aPBMCs cultured alone and after DPSCs co-culture with and without PD-L1 inib was evaluated by Western blot analyses. SC consisted in cleaved-caspase 3 positive control. *P < 0.05, **P < 0.01 ^§^*P* < 0.05 vs. aPBMCs alone

The interaction between PD-L1 and PD1 was shown in Fig. 3D and E when PD-L1-immunoprecipitated were revealed for PD1 and vice versa (Fig. 3D, E). Noteworthy, the interaction between PD-L1 and PD1 was clearly evident on either DPSCs and aPBMCs after co-culture, demonstrating that the interaction PD1/PD-L1 occurred in both stem cells and aPBMCs. Furthermore, we investigated the expression of PD1 and PD-L1 in CD4^+^ and CD4^−^ T lymphocytes in aPBMCs by flow cytometry (Gating strategy to determine PD1 and PD-L1 expression by aPBMCs was reported in Additional file 1: Fig. S2). Pre-activated T lymphocytes expressed PD-L1 on the cell surface while expressing PD1 at low levels. Expression was comparable between CD4^+^ and CD4^−^ T lymphocytes. Co-culture of aPBMCs with DPSCs did not modify PD1 and PD-L1 expression by T lymphocytes, whereas adding PD-L1 inhibitor during co-culture led to a significant decrease in PD-L1 expression by T lymphocytes (Fig. 4A; *P < 0.05 aPBMCs + PD-L1 inib; aPBMCs after DPSCs co-culture + PD-L1 inib vs. aPBMCs; ^§^*P* < 0.05, ^§§^*P* < 0.01 aPBMCs after DPSCs co-culture + PD-L1 inib vs. aPBMCs after DPSCs co-culture).

### Evaluation of inflammatory cytokines expression in PBMCs after DPSCs co-culture

The modulation of inflammatory status exerted by DPSCs was evaluated through RT-PCR analyses. In particular, mRNA levels of pro-inflammatory cytokines were investigated in aPBMCs cultured alone and after 72 h of co-culture with DPSCs. Resting PBMCs cultured alone were used as controls. As reported in Fig. 4B, pre-activation of PBMCs with CD3/CD28 antibodies sustained an inflammatory status resulting in the detection of pro-inflammatory cytokines in immune cells isolated from healthy donors (Fig. 4B; dark grey bars). Interestingly, aPBMCs after 72 h of co-culture with DPSCs expressed a significant reduction of IFNγ, TNFα, IL-2, IL-10, CCL5 and CXCL10 mRNA levels when compared to aPBMCs (^§§^*P* < 0.01, ^§§§^*P* < 0.001 vs. aPBMCs). The presence of PD-L1 inhibitor in the co-culture system did not restore mRNA levels of pro-inflammatory cytokines in aPBMCs showing low levels resembling those of co-culture without PD-L1 inhibitor (^§§^*P* < 0.01, ^§§§^*P* < 0.001 vs. aPBMCs). On the contrary, mRNA levels of IL-6 were significantly up-regulated in aPBMCs after co-culture with DPSCs (^§§§^P < 0.001 vs. aPBMCs). The use of PD-L1 inhibitor was able to down-regulate mRNA levels of IL-6 in aPBMCs after DPSCs co-culture compared to both aPBMCs alone and aPBMCs after DPSCs co-culture without PD-L1 inhibitor (^§§^*P* < 0.01 vs. aPBMCs, ^°°°^*P* < 0.01 vs. aPBMCs after DPSCs co-culture; Fig. 3B). These data indicate the correlation between PD-L1/PD1 axis and IL-6. As reported in Fig. 4B mRNA levels detected in aPBMCs after DPSCs co-culture, with and without PD-L1 inhibitor, resembled those of rPBMCs (*P < 0.05, **P < 0.01, ***P < 0.001 vs. rPBMCs). The immunosuppressive effects of DPSCs on aPBMCs were confirmed by analyzing the expression of caspase 3 through Western blot. After co-culture with DPSCs in presence or absence of PD-L1 inhibitor the expression of cleaved caspase 3 was significantly increased in comparison to aPBMCs cultured alone (*P < 0.05 vs. aPBMCs alone; Fig. 4C). These data are in accordance with Real Time PCR analysis (Additional file 1: Fig. S3). In parallel, the expression of PCNA was significantly reduced in aPBMCs after DPSCs co-culture with or without PD-L1 inhibitor (**P < 0.01 vs. aPBMCs alone; Fig. 4C).

### PD1/PD-L1 modulation in DPSCs under differentiation condition

**Fig. 5:**
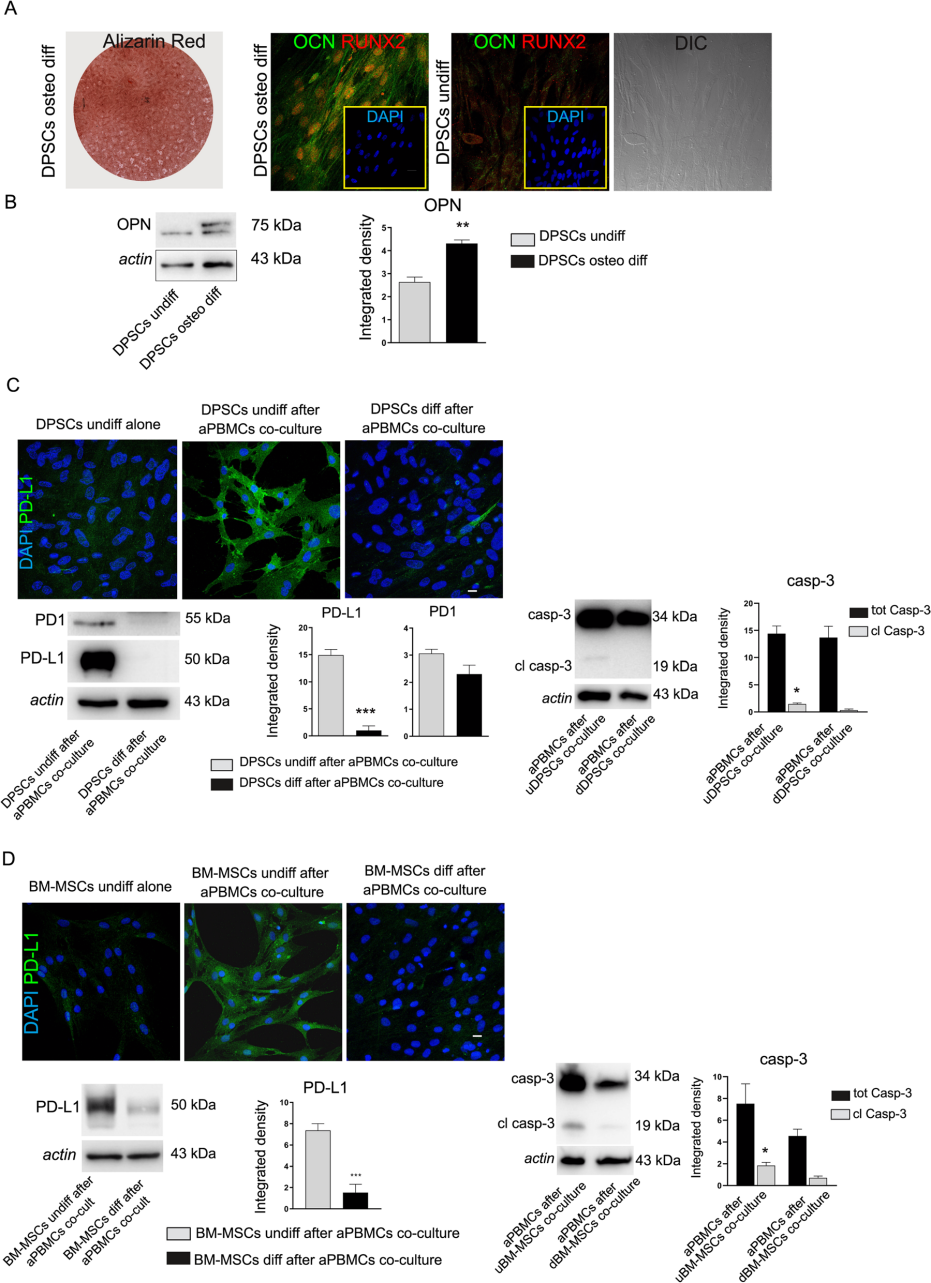
Modulation of PD1/PD-L1 pathway in differentiated DPSCs and BM-MSCs. **A** After 3 weeks of induction, osteogenic differentiation of DPSCs was demonstrated by deposition of mineralized extracellular matrix as revealed by Alizarin Red stain and confirmed by immunofluorescence analyses of OCN and RUNX2. Undifferentiated DPSCs were used as negative control. Nuclei were counterstained with DAPI (yellow square). DIC image of undifferentiated DPSCs was reported on the right. **B** Western blot analysis of OPN in undifferentiated DPSCs and in differentiated DPSCs. Histograms show a statistically significant increased expression of OPN in differentiated DPSCs. **P < 0.01 versus DPSCs undiff. **C** Immunofluorescence and Western blot analyses demonstrating the modulation of PD-L1 expression in DPSCs. Representative images showed undifferentiated DPSCs labeled strongly positive only after co-culture with aPBMCs, whereas, after reaching osteogenic commitment no PD-L1 expression was detected. Scale bar: 10 µm. Western blot analysis of PD1 and PD-L1 showed that PD-L1 expression is statistically significant decreased in differentiated DPSCs after co-culture with aPBMCs in comparison to undifferentiated DPSCs co-cultured with aPBMCs; ***P < 0.001 vs. DPSCs undiff after aPBMCs co-culture. On the right side Western blot analysis showing caspase 3 expression in aPBMCs after co-culture with undifferentiated DPSCs (uDPSCs) and differentiated DPSCs (dDPSCs); *P < 0.05 versus aPBMCs after dDPSCs co-culture. **D** Down-regulation of PD-L1 in osteogenic differentiated BM-MSCs after aPBMCs co-culture was revealed by Immunofluorescence and Western blot analyses. Histograms show that PD-L1 expression was statistically significant reduced in BM-MSCs; ***P < 0.001 vs. BM-MSCs undiff after aPBMCs co-culture. On the right side Western blot analysis showing caspase 3 expression in aPBMCs after co-culture with undifferentiated BM-MSCs (uBM-MSCs) and differentiated BM-MSCs (dBM-MSCs); *P < 0.05 versus aPBMCs after dBM-MSCs co-culture. For each statistical analysis, data were expressed as mean ± SD

DPSCs cultured in osteogenic medium for 3 weeks were able to differentiate towards osteogenic lineage, as demonstrated by Alizarin Red stain of mineralized extracellular matrix and confirmed by immunofluorescence analysis against the typical bone-related markers OCN and RUNX2. Undifferentiated DPSCs were used as negative control (Fig. 5A). Moreover, Western blot analysis of OPN further confirmed the achievement of osteogenic differentiation by DPSCs after 3 weeks of induction (**P < 0.01 vs. undifferentiated DPSCs; Fig. 5B). The immunomodulatory PD1/PD-L1 pathway was also investigated in differentiated stem cells. Particularly, confocal immunofluorescence analyses in Fig. 5C revealed that PD-L1 expression was down-regulated in osteogenic differentiated DPSCs after co-culture with aPBMCs, when compared to co-cultured undifferentiated DPSCs. These data were supported by Western blot analysis showing a statistically significant down-regulation of PD-L1 in co-cultured differentiated DPSCs (***P < 0.001 vs. DPSCs undiff after aPBMCs co-culture; Fig. 5C). At the same time, DPSCs induced towards osteogenic differentiation down regulated the expression of cell membrane receptor PD1 (Fig. 5C). These data suggest that PD1/PD-L1 dependent immunomodulatory properties of DPSCs are strictly related to their stemness phenotype and that, following cell differentiation, their expression is down-regulated. Noteworthy, PD-L1 down-regulation following osteogenic induction was also highlighted in BM-MSCs under the same culture conditions (***P < 0.001 vs. BM-MSCs undiff after aPBMCs co-culture; Fig. 5D). The evaluation of caspase 3 was carried out on aPBMCs after co-culture with undifferentiated stem cells (uDPSCs and uBM-MSCs) and with osteogenic differentiated stem cells (dDPSCs and dBM-MSCs). As reported in Fig. 5C, D, the expression of cleaved caspase 3 is significantly higher in aPBMCs after co-culture with undifferentiated stem cells when compared to aPBMCs after co-culture with differentiated stem cells (*P < 0.05 vs. aPBMCs after co-cultured with undifferentiated stem cells).

### Immunoregulatory function of DPSCs

**Fig. 6:**
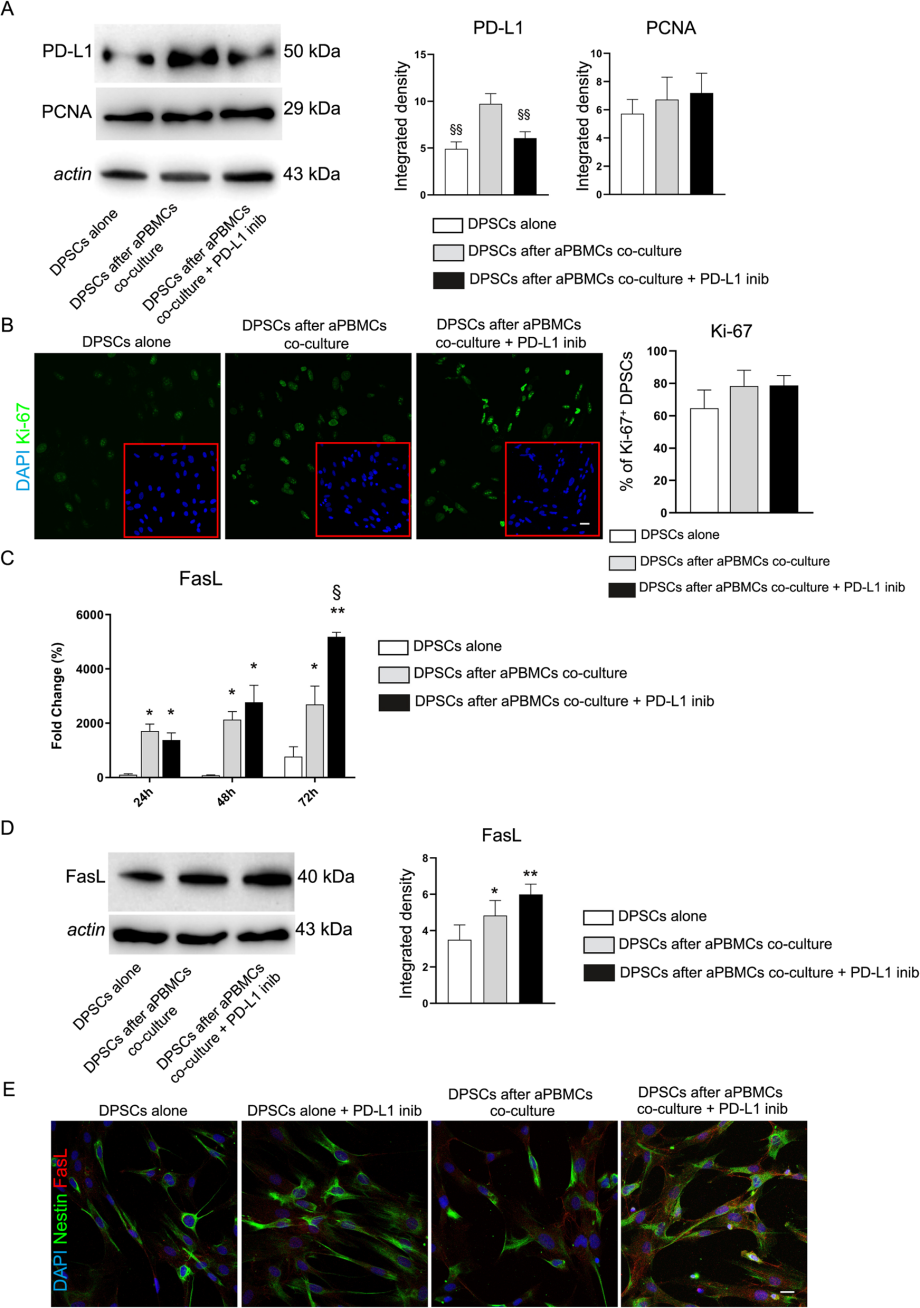
Immunoregulatory function of DPSCs. **A** Western blot analysis of PD-L1 and PCNA. A statistically significant reduction of PD-L1 expression was shown in DPSCs after aPBMCs co-culture + PD-L1 inib, similarly to DPSCs alone; ^§§^*P* < 0.01 vs. DPSCs after aPBMCs co-culture. **B** Evaluation of cell proliferation by Ki-67 immunofluorescence analysis. Histograms report the mean percentage of Ki-67^+^ DPSCs. Nuclei were counterstained with DAPI (red square). Scale bar: 10 µm. **C** Evaluation of FasL expression by Real Time PCR analyses revealed that after aPBMCs co-culture with and without PD-L1 inhibitor, DPSCs statistically significant up-regulated mRNA levels of FasL in a time-dependent manner; *P < 0.05, **P < 0.01 vs. DPSCs alone. At 72 h, a statistically significant up-regulation of FasL was detected in DPSCs after aPBMCs co-culture additioned with PD-L1 inhibitor versus DPSCs after aPBMCs co-culture; ^§^*P* < 0.05. **D** FasL expression was also evaluated by Western blot analysis in different experimental groups. Histograms reveal a statistically significant increase of FasL in DPSCs after aPBMCs co-culture with and without PD-L1 inib; *P < 0.05, **P < 0.01 vs. DPSCs alone. Data are represented as mean ± SD and one-way ANOVA followed by Newman-Keuls post-hoc test was carried out. **E** FasL expression (red) is confirmed by immunofluorescence analysis on Nestin^+^ DPSCs. Nuclei were counterstained with DAPI. Scale bar: 10 µm

In order to evaluate the immunoregulatory function of DPSCs a selective PD-L1 inhibitor was added to co-culture between DPSCs and aPBMCs. As shown in Fig. 6A, PD-L1 expression was statistically significant lower in DPSCs after co-culture with aPBMCs additioned with PD-L1 inhibitor, when compared to DPSCs after aPBMCs co-culture (Fig. 6A; ^§§^*P* < 0.01 vs. DPSCs after aPBMCs co-culture). At the same time, PD-L1 inhibitor did not induce any statistically significant alteration in DPSCs proliferation, as demonstrated by PCNA expression and percentage of Ki-67^+^ DPSCs among the experimental groups (Fig. 6A, B). Taken together, these data are in accordance with findings reported in Fig. 3B–E, which support the evidence that PD-L1 exerts effects on DPSCs, without affecting their proliferation.

Furthermore, the effects of PD-L1 inhibition on DPSCs immunosuppressive abilities were investigated analyzing the activation of Fas/FasL pathway. Real Time PCR analysis was carried out on DPSCs after 24, 48 and 72 h of co-culture aPBMCs with and without the addition of PD-L1 inhibitor. As shown in Fig. 6C, DPSCs after co-culture with aPBMCs revealed a statistically significant increase of FasL mRNA levels in a time dependent manner. When PD-L1 inhibitor was added to the co-culture, DPSCs maintained statistically significant high levels of FasL mRNA with respect to DPSCs alone. Interestingly, at 72 h of co-culture, PD-L1 inhibitor induced a further increase of FasL mRNA levels in DPSCs when compared to the counterpart co-culture without PD-L1 inhibitor (*P < 0.05, **P < 0.01 vs. DPSCs alone, ^§^*P* < 0.05 vs. DPSCs after aPBMCs co-culture). Based on these results, the expression of FasL was analyzed by Western Blot analysis after 72 h of co-culture. As reported in Fig. 6D, a statistically significant increase of FasL expression was detected in DPSCs after co-culture with aPBMCs in presence of PD-L1 inhibitor (Fig. 6D; *P < 0.05, **P < 0.01 vs. DPSCs alone). These data were also supported by confocal immunofluorescence analysis where the expression of FasL (red) was detected in Nestin^+^ DPSCs (green) (Fig. 6E). Based on this evidence, it may be argued that when PD-L1 inhibitor was added to the inflammatory microenvironment, a compensatory pathway was activated by DPSCs, in order to maintain their immunomodulatory abilities.

### PD-L1 modulation under inflammatory conditions

**Fig. 7:**
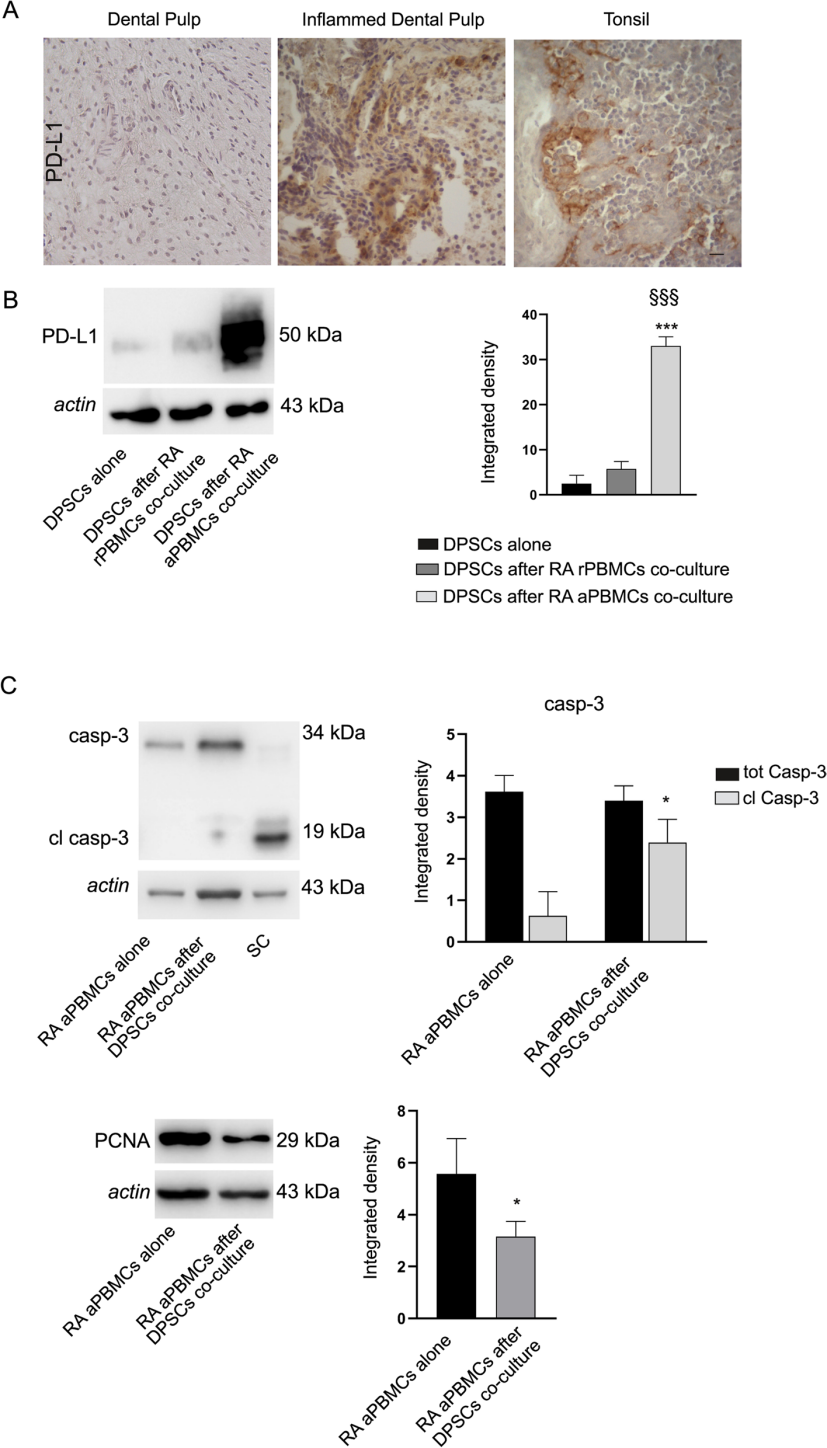
PD-L1 involvement in inflammatory microenvironment. **A** Immunohistochemical evaluation of PD-L1 in vivo revealing a strong labeling in pulpitis-affected dental pulp. Tonsil was used as a positive control. Scale bar: 50 µm. **B** PD-L1 expression was assayed by Western blot in DPSCs after co-culture with rPBMCs and aPBMCs isolated from rheumatoid arthritis patients (RA PBMCs). Histograms show a statistically significant higher expression of PD-L1 only in DPSCs after RA aPBMCs co-culture; ***P < 0.001 vs. DPSCs alone; ^§§§^*P* < 0.001 vs. DPSCs after RA rPBMCs co-culture. **C** Western blot analyses of caspase 3 and PCNA in RA aPBMCs alone and after co-culture with DPSCs. Histograms reveal statistically significant differences in cleaved caspase 3 and PCNA expression in RA aPBMCs after DPSCs co-culture; *P < 0.05 vs. RA aPBMCs alone. SC consisted in cleaved-caspase 3 positive control. Data are expressed as mean ± SD and analyzed by one-way ANOVA followed by Newman-Keuls post-hoc test

Immunohistochemistry analyses were performed on healthy and inflamed dental pulps. Interestingly, the presence of PD-L1 expressing cells was also detected in inflammatory status such as pulpitis, suggesting that up-regulation of PD-L1 does not rely only on allogeneic conditions but, even more importantly, may occur under autologous inflammatory conditions (Fig. 7A).

These data paved the way for the evaluation of PD-L1 expression in DPSCs after exposure to resting and pre-activated PBMCs isolated from RA patients. Indeed, the involvement of PD1/PD-L1 pathway in autoimmune diseases might be exploited as a new therapeutic tool of DPSCs. Preliminary results obtained through Western blot analyses revealed that DPSCs up-regulated PD-L1 also when co-cultured with RA aPBMCs (***P < 0.001 vs. DPSCs alone; ^§§§^*P* < 0.001 vs. DPSCs after RA rPBMCs co-culture; Fig. 7B) and, at the same time, induced activation of caspase 3 in RA aPBMCs when compared to RA aPBMCs cultured alone (*P < 0.05 vs. RA aPBMCs alone; Fig. 7C). To confirm the immunosuppressive effects of DPSCs on RA aPBMCs the expression of PCNA was evaluated by Western blot analysis. Histograms showed the statistically significant decrease of PCNA expression in RA aPBMCs after DPSCs co-culture (*P < 0.05 vs. RA aPBMCs alone).

## Discussion

Adult MSCs represent a new therapeutic tool in the field of regenerative medicine. MSCs primarily isolated from bone-marrow can be also obtained from different sources such as dental pulp and adipose tissue. Regarding dental pulp, it represents an interesting source of MSCs thanks to the peculiar origin from neural crest. Characteristics of DPSCs are not solely associated to the expression of typical mesenchymal surface markers and to the multi-differentiation potential toward different cell lineages (i.e., osteoblasts, chondroblasts, adipocytes) [1]. The low immunogenicity and the immunomodulatory functions represent key properties of DPSCs which make them promising candidates in cell-based therapy approaches to a variety of autoimmune and inflammatory diseases [14, 30, 31]. In fact, the therapeutic effects of MSCs may depend on their ability to regulate inflammation for favoring tissue homeostasis, replacement and regeneration. As well-established, the immunomodulatory ability of stem cells is highly plastic and depends upon the kind and concentration of inflammatory mediators which orchestrate the inflammatory microenvironment supporting the hypothesis that MSCs can (1) exert an immunosuppressive role or (2) empower the immune response [18, 32, 33]. The inflammatory microenvironment could also influence the activation of immunomodulatory mechanisms exerted by MSCs [11, 18, 34, 35]. Although the mechanisms by which DPSCs exert their immuno-regulatory functions have been widely investigated, they have not been fully elucidated yet. Among direct and indirect mechanisms implicated, Fas/FasL pathway has been demonstrated to play a pivotal role in DPSCs-mediated immunomodulation and in the maintenance of stemness properties [12, 14]. In the present study we demonstrated that the creation of an inflammatory microenvironment in vitro*,* following direct or indirect interaction of stem cells with immune cells, elicits the activation of PD-L1 in DPSCs. It is well known that the engagement of PD-L1 with PD1 regulates T cell response and it is important to maintain the balance between peripheral tolerance and autoimmunity [23, 36]. PD1 is also expressed in other cytotypes such as retinal ganglion cells and is required to maintain stem cell properties in neural crest derived DPSCs also suggesting its engagement in the osteogenic differentiation [22]. On the other hand, the role of PD-L1 in DPSCs is not fully understood. Our data point out that DPSCs up-regulate PD-L1 via both direct and indirect interaction-dependent mechanisms in response to exposure to CD3/CD28 co-stimulated PBMCs. Our data demonstrated that these properties are not exclusive features of DPSCs but are also shared by AFSCs and BM-MSCs suggesting that PD-L1 is an immunomodulatory checkpoint exploited by stem cells when exposed to inflammatory stimuli. These data are in accordance with previous reports and among the inflammatory cytokines, IFNγ and TNFα synergistically induce the stimulation and the immune-regulatory activation of MSCs [37–39]. Indeed, pre-activation with CD3/CD28 induces an increase in mRNA levels of IFNγ, TNFα, IL-2, IL-10, IL-6, CCL5 and CXCL10 in PBMCs prior to co-culturing with stem cells. Several findings have highlighted that IFNγ plays a pivotal role in the induction and expression of PD-L1 in MSCs [40, 41]. Interestingly, after co-cultures mRNA levels of pro-inflammatory cytokines in PBMCs were inhibited. Regarding IL-2, it is well known that its downregulation is linked to T cells anergy, a mechanism already investigated in T lymphocytes after MSCs co-culture [42–44]. Noteworthy, our results revealed that after co-culture with DPSCs, the intracellular levels of chemotactic chemokines CXCL10 and CCL5 in aPBMCs were downregulated. CXCL10 and CCL5 hold a prominent role in T cell recruitment and migration during inflammatory diseases [45]. Our results are in accordance with findings from Davies et al. showing that the selective blockade of PD-L1 in co-culture conditions did not reverse the downregulation of above-mentioned cytokines [46]. This event might be due to the activation of compensatory pathways that synergistically allow DPSCs to modulate the inflammatory microenvironment. Regarding IL-10, the co-culture with DPSCs induced a reduction of mRNA levels in aPBMCs. These data are in accordance with our previous findings [15] and further investigations are needed to better understand the pleiotropic effect of IL-10. On the other hand, the increased IL-6 levels in aPBMCs after DPSCs co-culture are suppressed in the presence of the specific PD-L1 inhibitor, suggesting that a tight correlation between PD1/PD-L1 and IL-6 axis exists and might be functional also in DPSCs [15, 47, 48]. Based on these results, DPSCs not only exert an immunomodulatory effect, but also own an anti-inflammatory activity that can be fulfilled through a concert of different mechanisms recruited during the progression of the inflammatory process. Furthermore, our data highlighted that the selective PD-L1 inhibitor induced the up-regulation of FasL in DPSCs, whose signaling in stem cells is renowned to trigger apoptosis in immune cells [12, 13]. Our data suggest that PD-L1 produced by DPSCs is not only functional in controlling immune response but is also exploited by DPSCs themselves to maintain their immunomodulatory properties. On these bases, stem cells may take advantage of the crosstalk existing among different immunomodulatory pathways which share the common aim to control the immune response and thus achieve tissue homeostasis by creating favorable conditions for tissue repair and regeneration [18]. To this regard, we also highlighted that PD-L1 expression is recruited during an inflammatory status such as pulpitis, prompting that the expression of PD-L1 is not simply due to the allogeneic co-culture system but is an anti-inflammatory/immunomodulatory mechanism exerted by DPSCs. As a matter of fact, after reaching the osteogenic commitment, the differentiated DPSCs co-cultured with aPBMCs lose the capability to express PD-L1, hinting that this feature is strictly correlated with the stemness status of DPSCs. In conclusion, since PD1/PD-L1 pathway plays a pivotal role in immune tolerance whose failure is characteristic in the establishment and development of autoimmune diseases such as rheumatoid arthritis it would be interesting to evaluate the efficacy of PD-L1-mediated anti-inflammatory potential of DPSCs when exposed to a chronic inflammatory microenvironment [9]. Our preliminary data confirmed that DPSCs are able to up-regulate PD-L1 after co-culture with RA aPBMCs and according to Guo et al. the agonist ligand of PD1 might represent a new therapeutic tool in the treatment of RA and preventing the progression to RA [49].

## Conclusions

In the present study, we demonstrated that DPSCs can modulate the inflammatory microenvironment by the activation of PD1/PD-L1 pathway synergistically cooperating with other immune-regulatory pathways including Fas/FasL. In light of this evidence, these properties might be functional in controlling the inflammatory milieu typical of autoimmune diseases, such as rheumatoid arthritis. To this regard, the present study provided new insights on immunoregulatory functions of DPSCs that, corroborated by further investigations, might be exploited to develop novel therapeutic tools in the treatment of autoimmune diseases.

## Supplementary Information

**Additional file 1: Figure S1. Gating strategy to determine PD-1 and PD-L1 expression by DPSCs.** DPSCs were identified by FSC and SSC (1) then live cells were gated by SSC/dead staining dot plot (2). Afterwards CD3neg CD4neg cells were selected (3) and fluorescence intensities in FITC and PECy7 channels shown by histograms. The mean percentage of adherent DPSCs in the FSC/SSC gating was 93.5% ± 2.2%. **Figure S2. Gating strategy to determine PD-1 and PD-L1 expression by PBMCs pre-activated by CD3/CD28 linking.** PBMCs were identified by FSC and SSC (1) then live cells were gated by SSC/dead staining dot plot (2). Afterwards CD3+CD56neg cells (T lymphocytes) were selected (3). CD4+ and CD4neg cells were further gated (4) and fluorescence intensities in FITC and PECy7 channels shown by histograms. **Figure S3. Real Time PCR analysis of caspase 3 in PBMCs.** Caspase 3 mRNA levels were evaluated in rPBMCs and aPBMCs alone, aPBMCs after DPSCs co-culture with and without PD-L1 inhibitor. Statistically significant increase of mRNA levels of caspase 3 was detected in aPBMCs after DPSCs co-culture with and without the addition of PD-L1 inhibitor (^§^P < 0.05 vs aPBMCs).

## Data Availability

All data generated or analysed during this study are included in this article and its Additional file 1.

## Abbreviations

DPSCs: Dental pulp stem cells
MSCs: Mesenchymal stem cells
PBMCs: Peripheral blood mononuclear cells
PD-L1: Programmed cell death-ligand 1
PD1: Programmed cell death 1
FBS: Fetal bovine serum
FACS: Fluorescence activated cell sorting
PFA: Paraformaldehyde
AFSCs: Amniotic fluids stem cells
BM-MSCs: Bone marrow mesenchymal stem cells
OPN: Osteopontin
PBS: Phosphate buffer saline
BSA: Bovine serum albumine
OCN: Osteocalcin
DAPI: 4',6-Diamidino-2-phenylindole, dihydrochloride
DAB: 3,3’-Diaminobenzidine
rPBMCs: Resting PBMCs
aPBMCs: Pre-activated PBMCs
PD-L1 inib: PD-L1 inhibitor
RA: Rheumatoid arthritis
