# Supplementary material for: Role of PD-L1 in licensing immunoregulatory function of dental pulp mesenchymal stem cells
Source: Stem Cell Res Ther. 2021 Dec 4;12:598. doi: 10.1186/s13287-021-02664-4 (PMC8643194; doi:10.1186/s13287-021-02664-4)

**Additional file 1**

**Role of PD-L1 in licensing immunoregulatory function of dental pulp mesenchymal stem cells**

Rosanna Di Tinco^1*^, Giulia Bertani^1*^, Alessandra Pisciotta^1^, Laura Bertoni^1^, Elisa Pignatti^1^,Monia Maccaferri^1^, Jessika Bertacchini^1^, Paola Sena^1^, Antonio Vallarola^2^, Rossella Tupler^2^, Stefania Croci^3^, Martina Bonacini^3^, Carlo Salvarani^1,4^, Gianluca Carnevale^1§^

^1^Department of Surgery, Medicine Dentistry and Morphological Sciences with Interest in Transplant, University of Modena and Reggio Emilia, Modena, Italy.

^2^Department of Biomedical, Metabolic and Neural Sciences, Center for Neuroscience and Neurotechnology, University of Modena and Reggio Emilia, Modena, Italy

^3^Clinical Immunology, Allergy and Advanced Biotechnologies Unit, Azienda Unità Sanitaria Locale-IRCCS di Reggio Emilia, Reggio Emilia, Italy.

^4^Rheumatology Unit, Azienda Unità Sanitaria Locale-IRCCS di Reggio Emilia, Reggio Emilia, Italy.

**Supplementary Figure 1. Gating strategy to determine PD-1 and PD-L1 expression by DPSCs.**

DPSCs were identified by FSC and SSC (1) then live cells were gated by SSC/dead staining dot plot (2). Afterwards CD3negCD4neg cells were selected (3) and fluorescence intensities in FITC and PECy7 channels shown by histograms. The mean percentage of adherent DPSCs in the FSC /SSC gating was 93.5% ± 2.2%.


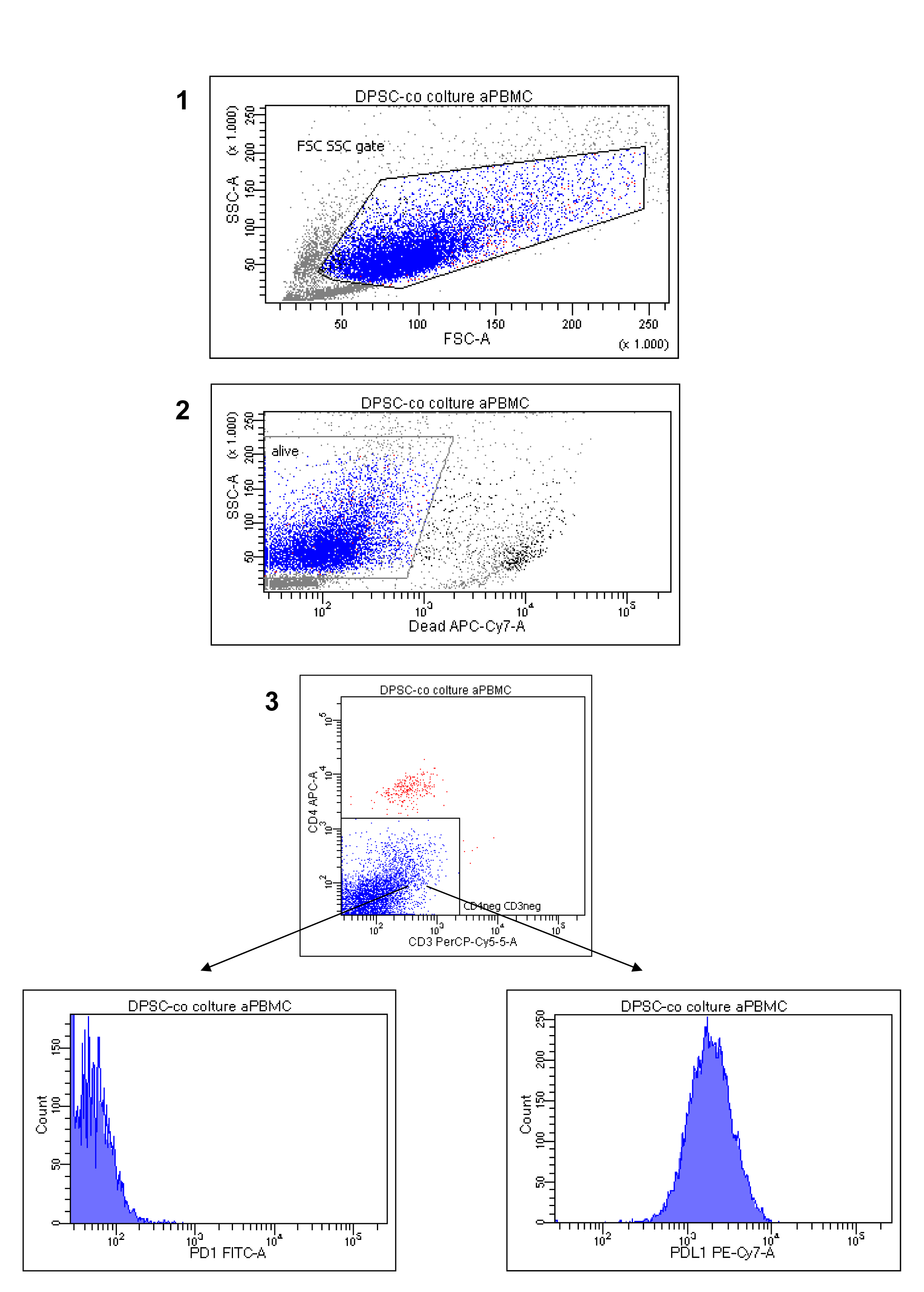


**Supplementary Figure 2. Gating strategy to determine PD-1 and PD-L1 expression by PBMCs pre-activated by CD3/CD28 linking.**

PBMCs were identified by FSC and SSC (1) then live cells were gated by SSC/dead staining dot plot (2). Afterwards CD3+CD56neg cells (T lymphocytes) were selected (3). CD4+ and CD4neg cells were further gated (4) and fluorescence intensities in FITC and PECy7 channels shown by histograms


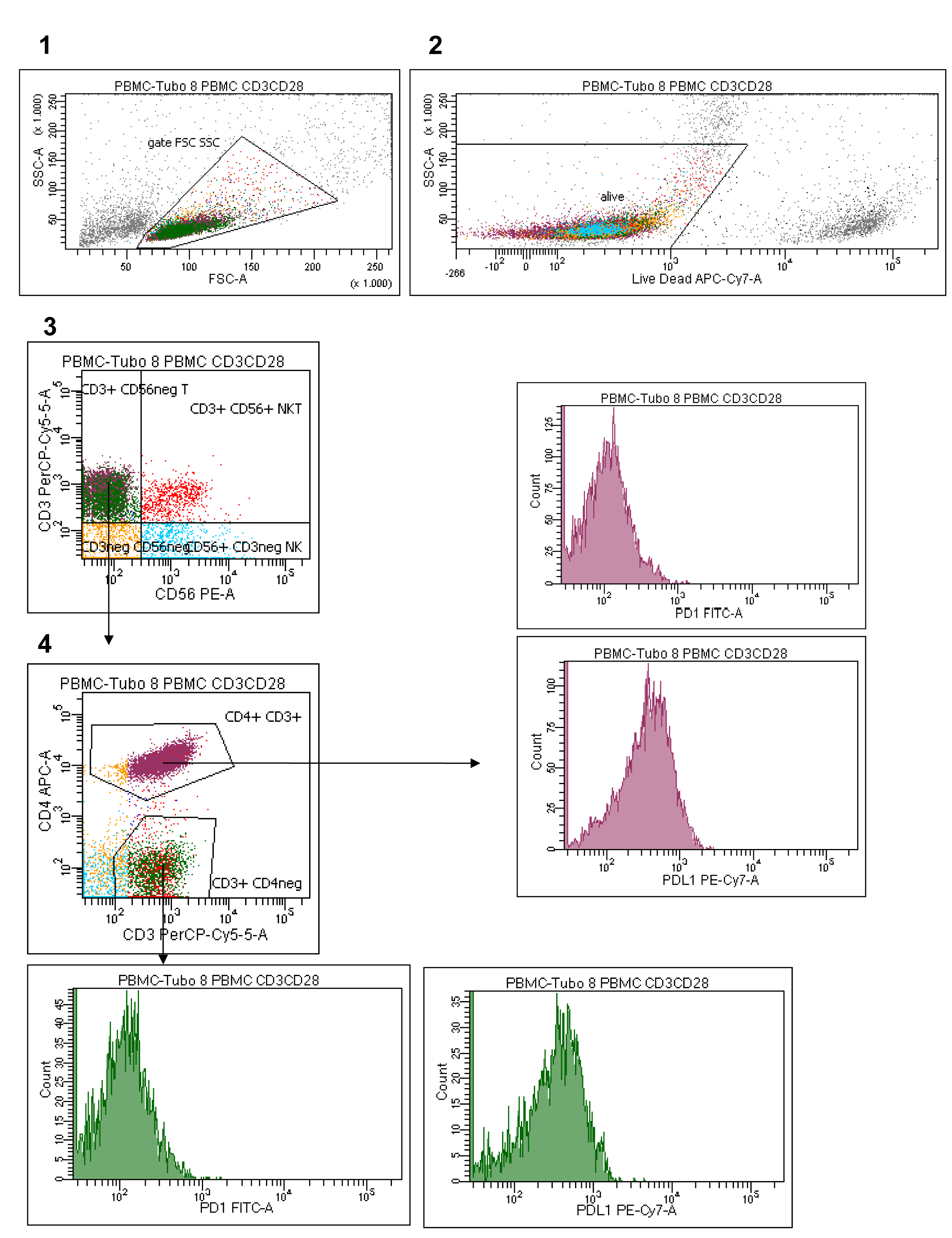


**Supplementary Figure 3. Real Time PCR analysis of caspase 3 in PBMCs.** Caspase 3 mRNA levels were evaluated in rPBMCs and aPBMCs alone, aPBMCs after DPSCs co-culture with and without PD-L1 inhibitor. Statistically significant increase of mRNA levels of caspase 3 was detected in aPBMCs after DPSCs co-culture with and without the addition of PD-L1 inhibitor (^§^*P*<0.05 vs aPBMCs).


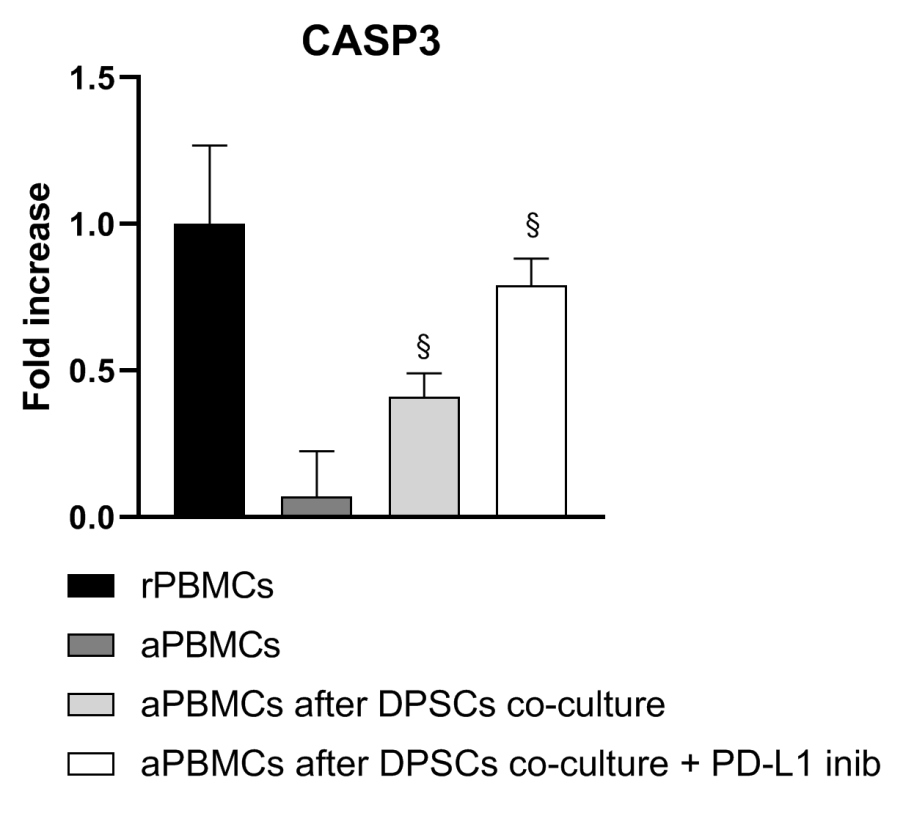

Supplement: Supplementary file 1 — Additional file 1: Figure S1. Gating strategy to determine PD-1 and PD-L1 expression by DPSCs. DPSCs were identified by FSC and SSC (1) then live cells were gated by SSC/dead staining dot plot (2). Afterwards CD3neg CD4neg cells were selected (3) and fluorescence intensities in FITC and PECy7 channels shown by histograms. The mean percentage of adherent DPSCs in the FSC/SSC gating was 93.5% ± 2.2%. Figure S2. Gating strategy to determine PD-1 and PD-L1 expression by PBMCs pre-activated by CD3/CD28 linking. PBMCs were identified by FSC and SSC (1) then live cells were gated by SSC/dead staining dot plot (2). Afterwards CD3+CD56neg cells (T lymphocytes) were selected (3). CD4+ and CD4neg cells were further gated (4) and fluorescence intensities in FITC and PECy7 channels shown by histograms. Figure S3. Real Time PCR analysis of caspase 3 in PBMCs. Caspase 3 mRNA levels were evaluated in rPBMCs and aPBMCs alone, aPBMCs after DPSCs co-culture with and without PD-L1 inhibitor. Statistically significant increase of mRNA levels of caspase 3 was detected in aPBMCs after DPSCs co-culture with and without the addition of PD-L1 inhibitor (§P < 0.05 vs aPBMCs). [file 13287_2021_2664_MOESM1_ESM.docx]
